# Supplementary material for: Functional characterization of extrinsic tongue muscles in the Pink1-/- rat model of Parkinson disease
Source: PLoS One. 2020 Oct 16;15(10):e0240366. doi: 10.1371/journal.pone.0240366 (PMC7567376; doi:10.1371/journal.pone.0240366)
Supplement: S1 Raw images — (PDF) [file pone.0240366.s001.pdf]

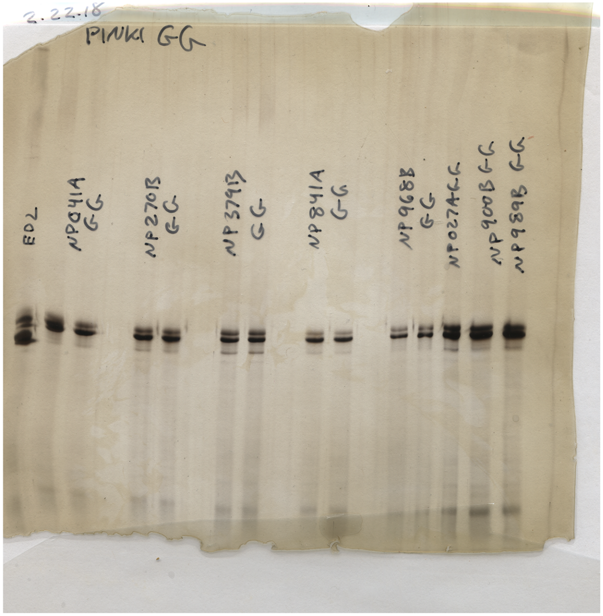

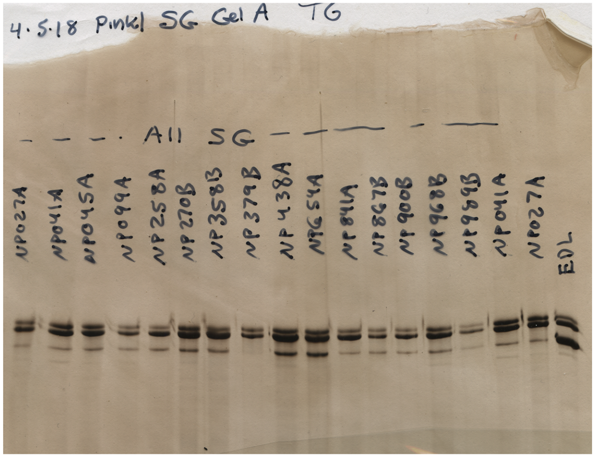

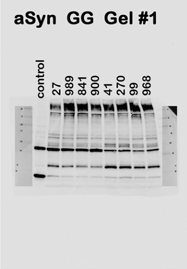

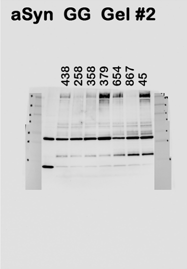

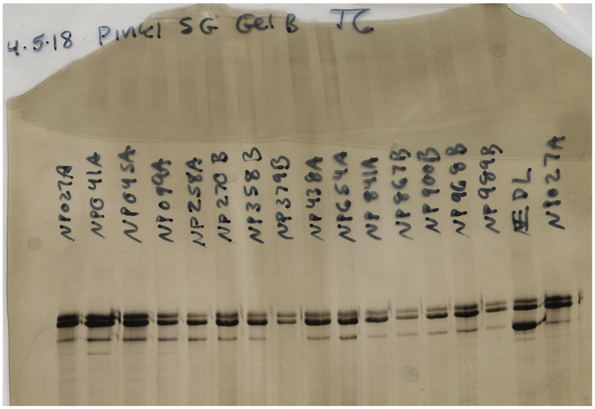

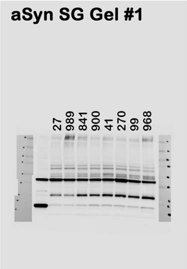

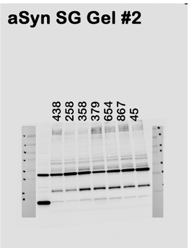

[redacted]

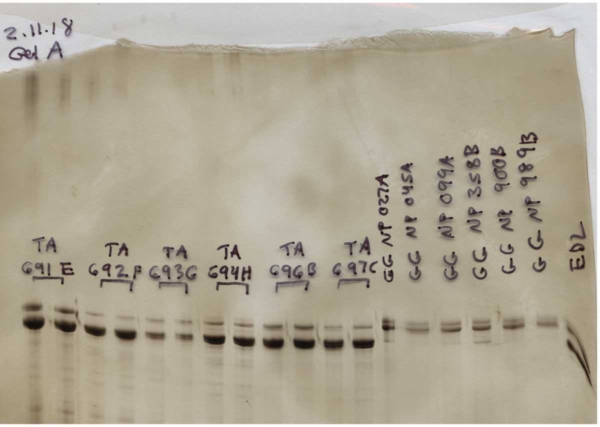

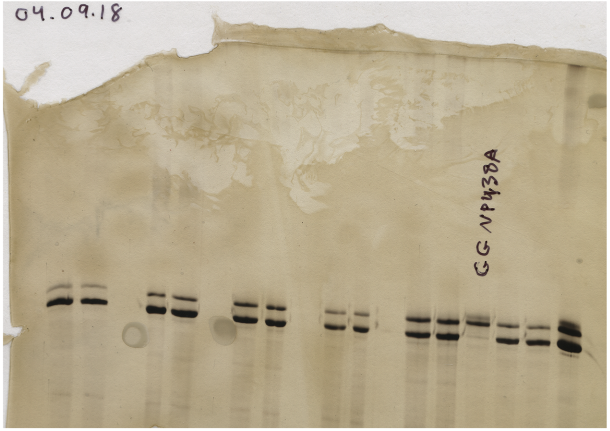

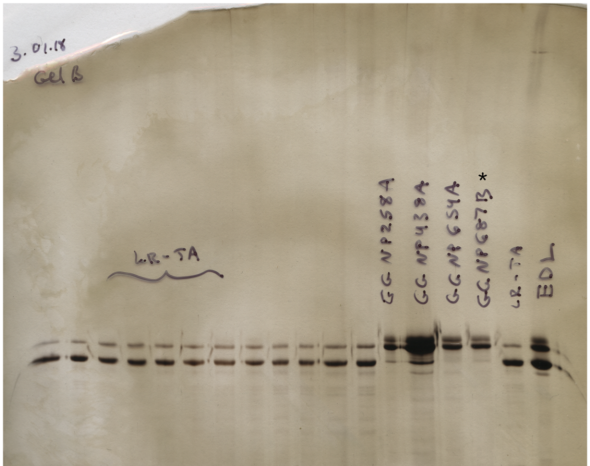

Note for gels: Samples for multiple studies are sometimes run on one gel. In those cases, samples unrelated to this manuscript are unlabeled here, or are labeled with codes that are not included in the Sample Key.

GG: Genioglossus

SG: Styloglossus

* = written label error; sample ID: NP867B

Sample Key

Animal ID	Genotype	Sac time point
NP654A	PINK1(-/-)	6 mo
NP867B	PINK1(-/-)	6 mo
NP045A	PINK1(-/-)	6 mo
NP379B	PINK1(-/-)	6 mo
NP438A	WT	6 mo
NP811B	WT	6 mo
NP258A	WT	6 mo
NP358B	WT	6 mo
NP041A	PINK1(-/-)	6 mo
NP270B	PINK1(-/-)	6 mo
NP099A	PINK1(-/-)	6 mo
NP968B	PINK1(-/-)	6 mo
NP027A	WT	6 mo
NP989B	WT	6 mo
NP841A	WT	6 mo
NP900B	WT	6 mo
